# Southern Dental Association Meeting

**Published:** 1882-06

**Authors:** J. B. Hodgkin


J. B. HODGKIN.

---

*Southern Dental Association.*—We again call the attention of the profession to the coming meeting of this Association in Baltimore on the 8th of August next. From many points come letters stating the intention of the writers to be present, and a useful and pleasant meeting is anticipated. One point we wish to correct. It is intimated by a letter writer that this is an Association promotive of a continuance of sectional animosity. Not so: This is a big country, and can hold many Associations as large as the Southern Dental and American Dental; and as the latter usually holds its meetings not far from the Canada line, the Southern Dentists should have an Association meeting in such localities that they can attend it at a reasonable cost of time and money. The Southern Dental was in the foremost rank in the formation of a national Association, which is broad enough and liberal enough to receive all reputable dentist, of whatever locality or shade of belief.

J. B. Hodgkin.

